# Death Following the Inhalation of Nitrous Oxide Gas

**Published:** 1873-04

**Authors:** 


### EDITORIAL, ETC.

*Death Following the Inhalation of Nitrous Oxide Gas.*—The February number of the "British Journal of Dental Science" contains the following account of a serious result from the administration of Nitrous Oxide, which occurred at the office of J. Thomas Brown Mason, Exeter, England, on January 22d, 1873. A coroner's jury rendered a verdict of "Homicide by Misadventure," and at the same time exonerated the dental practitioner by expressing the opinion that every necessary precaution was taken by Mr. Mason in administering the gas:

"The recent sad death at Exeter, coincident with the administration of nitrous oxide for the extraction of a tooth, while demanding from all the greatest sympathy for the friends of the unfortunate patient, cannot but excite in the minds of his professional brethren a deep feeling of regret for the gentleman whose lot it has been to witness such a scene in his surgery. The more the particulars of the case are examined, the more it must be felt by all, that at any moment a similar case may befall any one of us in the fulfilment of our professional duties. The patient apparently in full health and vigor—the operator experienced in the administration of the gas—of acknowledged skill in the use of his instruments—the relative and medical adviser of the patient in attendance—a practitioner of repute on the spot within three minutes of the occurrence—every element of safety apparently present—and yet in a few brief minutes all is sadly over, and by the unfortunate omission of post-mortem examination, we are left to vague conjectures as to the cause of death—or bold assertions, which are as unprofessional as they are mischievous. In the absence of direct evidence we have no more right to call this a death from nitrous oxide than we should have so to designate a similar case which happened in America, but which a post-mortem showed was due to a cork slipping into the throat.

"We remember one case where a patient called on a practitioner and requested to have chloroform at once for the extraction of a tooth, but was induced to wait for an appointment. The next day the dentist who was to have operated, saw by the morning papers that his patient on the way home, after leaving him, had dropped dead on the railway platform. Had an anæsthetic been administered at the time first desired, the patient would probably have then died, and the blame would have been laid to the agent used. We have ourselves seen two, apparently all but fatal cases, in which the danger was due, in the one instance, to an accumulation of phlegm in the throat—the result of whooping-cough—and in the other to tonsils so enlarged (it was afterwards found) as to cause dangerous distress during natural sleep.

"On reading over the evidence delivered at the inquest on this alleged death from nitrous oxide at Exeter, we fail to see why nitrous oxide should in this particular instance have been fatal. The second inhalation was not commenced until the patient had recovered from the first three or four inspirations sufficiently to speak rationally. The second inhalation, in view of a difficult operation, was probably pushed further than for an ordinary extraction, but there is no reason to suppose that it was continued beyond the point to which experience shows it can be safely carried. Even if the breathing was temporarily arrested, the fact that it was restored while the pulse was strong enough to be felt in the wrist, seems to show that something besides the gas must have caused death; for the recovery of the color of the blood is so immediate on breathing pure air, that some return of natural color ought to have been produced.

"It is somewhat remarkable that neither at the inquest nor in any subsequent remarks upon the case, is any mention made of the use of artificial respiration, but we understand from Mr. Mason that this measure was promptly and efficiently adopted by the medical men in attendance.

"It has been suggested that blood might have dropped into the trachea, but this would probably have given rise to some rattling noise, which would have been loud enough to have been noticed by the doctors.

" Is it not possible that an attack of apoplexy, arising from the bursting of an aneurism at the base of the brain, occurred about the time of commencing the second inhalation, and that the pressure of blood upon the brain and medulla oblongata put a stop to the respiratory movements.

" In this instance, however, the mode of death was more rapid than is usual in such cases, but we have been informed of a case where gas was intended to have been given to a patient who, owing to the absence of the administrator, submitted to extraction without taking any anæsthetic. She was observed to have the mouth drawn on one side when the operation was concluded. This paralysis extended to apoplexy, of which she shortly died—a vessel within the cranium having burst and poured forth blood, which caused death by its pressure upon the brain. A rupture of the heart or of an internal aneurism is a possibility at least as likely as that the death was occasioned by nitrous oxide administered in the manner stated in evidence. If 150,000 persons of all ages can take gas without a fatal result, and if, when animals seem on the point of death, they are restored by artificial respiration, it is surely likely that some unsuspected internal weakness existed in the present case, to which death ought in justice to be attributed.

" It is deeply to be regretted that a post-mortem examination was not insisted upon by the coroner. It seems to have been taken for granted that death, following so quickly upon the administration of the gas, must have been caused by it, as no other cause was evident. If heart disease had been diagnosed previously, and no anæsthetic had been given, death would have been attributed to heart disease; but there are various forms of disease of the heart and the large vessels which would escape detection if not specially sought for, and the fact of no such disease having been discovered, should not have been allowed to satisfy the jury.. A post-mortem examination can alone clear up the mystery, and we hope that the friends of the deceased will, in the interests of suffering humanity, allow the body to be exhumed for that purpose."

